# 免疫检查点抑制剂在小细胞肺癌中的临床研究进展

**DOI:** 10.3779/j.issn.1009-3419.2017.09.06

**Published:** 2017-09-20

**Authors:** 爽 张

**Affiliations:** 130012 长春，吉林省肿瘤医院胸部肿瘤内科 Department of Thoracic Oncology, Jilin Provincial Cancer Hospital, Changchun 130012, China

**Keywords:** 小细胞肺癌, 免疫检查点, 免疫检查点抑制剂, Small cell lung cancer, Immune checkpoint, Immune checkpoint inhibitor

## Abstract

小细胞肺癌（small cell lung cancer, SCLC）是分化较差的高级别肺神经内分泌肿瘤，尽管仅占所有肺癌的14%左右，但生长迅速、较早出现转移，复发后缺少有效的治疗手段，改善SCLC治疗迫在眉睫。近年来，肿瘤免疫治疗展现了良好的前景，尤其程序性死亡受体1（programmed death 1, PD-1）和细胞毒性T淋巴细胞相关抗原4（cytotoxic T-lymphocyte-associated antigen 4, CTLA-4）抑制剂的研究正在改变多种实体瘤的临床实践。SCLC与吸烟密切相关，具有较高的肿瘤突变负荷，是免疫治疗潜在理想的肿瘤类型。本文将总结免疫治疗在SCLC的临床研究进展，探讨SCLC免疫治疗中存在的问题、面临的挑战以及未来的应用前景。

## Abstract (en)

Small cell lung cancer (SCLC) is a poorly differentiated high-grade neuroendocrine tumor, accounts for approximately 14% of all lung cancers. SCLC is characterized by rapid growth, early metastasis without effective treatments after recurrence. It is urgently need to improve the therapy of patients with SCLC. In recent years Tumor immunotherapy has shown promising efficacy, especially in immune checkpoints including inhibitors programmed cell-death protein 1 (PD-1) and cytotoxic T-lymphocyte-associated protein 4 (CTLA-4). These immune checkpoint inhibitors of the researches are changing the clinical practice of many kinds of solid tumor. SCLC is a potential ideal type of tumor immunotherapy for tobacco exposure and the highest mutational load. In this report, the authors review the current state of the immunotherapy in SCLC, to discussing the problems, challenge and application development prospect.

小细胞肺癌（small cell lung cancer, SCLC）是肺癌中侵袭性最强的亚型，约占肺癌的14%，SCLC肿瘤倍增时间短，病情进展迅速，早期出现转移，尽管SCLC对放化疗敏感，但复发耐药不可避免的发生，而且疾病进展后缺少有效的治疗手段，因此改善SCLC的治疗一直是临床面临的棘手的问题。肿瘤的免疫治疗经历100多年的沉浮，2011年随着首个免疫检测点抑制剂Ipilimumab的获批，迎来了新一轮的研究热潮，免疫检查点阻滞治疗作为免疫治疗中的重要的一员，成为肿瘤治疗领域最耀眼的明星，在多种肿瘤中都有不俗表现。短短几内年除了ipilimumab，还有Nivolumab、Pembrolizumab、Atezolizumab、Avelumab相继上市，为晚期癌症患者带来了希望。而一向对各种治疗都波澜不惊的SCLC，在肿瘤免疫治疗的热潮下也泛起阵阵涟漪。

## 1 免疫检查点抑制剂用于SCLC的临床研究进展

### 1.1 CTLA-4抑制剂

SCLC与吸烟密切相关，基因组不稳定，突变负荷高^[1]^，理论上存在较高的肿瘤抗原，可能是免疫治疗的理想肿瘤类型。首先涉足SCLC的免疫靶向治疗是CTLA-4抑制剂Ipilimumab，CA184-041 Ⅱ期研究初步显示广泛期小细胞肺癌（extensive-stage small cell lung cancer, ED-SCLC）Ipilimumab联合化疗较化疗获得免疫相关无进展生存时间（immune-related progression-free survival，irPFS）的改善^[2]^。然而进一步的Ⅲ期研究CA184-156研究^[3]^并没有进一步验证Ⅱ期研究的结果，Ipilimumab联合标准化疗并不能够改善ED-SCLC的总生存时间（overall surviva, OS）和无进展生存时间（progression-free survival, PFS），分析其原因可能为：Ipilimumab联合标准化疗并没有引起肿瘤微环境中T细胞的活化，不能有效的激起足够的抗肿瘤免疫；伴随的化疗可能限制了T细胞的活化和增殖。

### 1.2 PD-1抑制剂

PD-1抑制剂Nivolumab以双免疫检查点联合抑制开启SCLC的临床研究。死亡受体-1/死亡受体配体-1（programmed death-1/PD-1 ligand 1, PD-1/PD-L1）和CTLA-4抑制剂通过不同的途径发挥免疫抑制作用，分别作用于T细胞活化的不同阶段，两个途径的联合抑制将更有效的发挥抗肿瘤免疫作用。2015年美国临床肿瘤学会年会（American Society of Clinical Oncology, ASCO）上Nivolumab±Ipilimumab治疗复发耐药SCLC的Ⅰ期CheckMate 032研究^[4]^让人们看到SCLC进行免疫靶向治疗的希望。2016年相关结果在*Lancet Oncol*^[5]^发布，研究认为对于复发耐药SCLC，Nivolumab单药和Nivolumab联合Ipilimumab治疗具有持久的抗肿瘤活性，尽管联合治疗毒性发生率更高但易于管理，联合治疗的生存结果令人鼓舞，值得一提的是，CheckMate 032研究有超过一半的患者既往接受2个方案以上治疗，与既往拓扑替康或者氨柔比星单药治疗复发耐药SCLC的历史对照研究相比1年的生存率相当甚至更优^[6, 7]^。2016年世界肺癌大会（World Conference on Lung Cancer, WCLC）发布了该研究2年的生存率^[8]^，联合治疗组达30%。美国国家综合癌症网络（National Comprehensive Cancer Network, NCCN）指南2017年第一版将Nivolumab±Ipilimumab（Nivo±Ipi）作为复发SCLC二线治疗的推荐方案之一^[9]^，SCLC的临床实践将随着这项临床研究而改变。2017年ASCO报道了该研究的后续结果^[10]^，Ⅰ期研究非随机队列部分由独立中心评审（blinded independented central review, BICR）评估的结果，联合治疗组的客观缓解率（objective response rate, ORR）为23%，2年OS率为26%，中位的OS为7.8个月，单药组，ORR为11%，2年OS率为14%，中位的OS为4.1个月；对非随机队列116例患者的PD-L1表达情况分析发现，SCLC中PD-L1≥1%的患者占18%，PD-L1表达与疗效并无相关性。Ⅱ期随机部分，联合治疗组和单药治疗组的ORR分别为21%和12%，3个月的PFS率分别为30%和18%，3个月的OS率分别为64%和65%，非随机队列部分和随机部分的汇集分析发现，接受联合治疗的患者与单药患者的ORR分别为22%和11%，毒性方面：联合治疗3级-4级治疗相关毒性的发生率是78%，单药治疗组是45%，联合治疗明显高于单药治疗，尤其是在皮肤毒性、肝功能、胃肠道毒性和内分泌毒性等方面，3级-4级毒性中位恢复时间是1.8周-16.3周。该研究进一步证实Nivo±Ipi方案的优势。然而在关注疗效的同时，如何兼顾联合治疗的毒性，用药剂量、用药持续时间，怎样合理搭配是需要探索的问题。CheckMate 032研究只是比较不同免疫治疗方案对复发耐药SCLC的疗效，并没有与目前标准的二线化疗方案进行比较，CheckMate 331研究则头对头比较了Nivo与拓扑替康或者氨柔比星对一线治疗后复发SCLC的疗效，这项研究结果将对SCLC二线治疗的选择产生重要影响。Nivo在SCLC维持治疗领域也进行了探索，正在进行的Check Mate 451研究Ⅲ期，比较Nivo单药、Nivo+Ipi、安慰剂在ED-SCLC维持治疗的疗效，将回答SCLC是否进行免疫维持治疗以及选择哪种免疫治疗维持方案的问题。另外，Nivo±Ipi联合针对SCLC神经内分泌分化的抗体偶联药物Rovalpituzumab治疗复ED-SCLC的Ⅰ期/Ⅱ期研究正在进行（NCT03026166），这也将开启免疫靶向治疗与SCLC发生发展相关靶点的研究。

另一个PD-1抑制剂Pembrolizumab在SCLC领域也崭露头角。KEYNOTE-028研究1b期^[11]^通过22C3抗体筛选肿瘤细胞≥1%细胞膜性PD-L1阳性的经标准治疗失败的SCLC患者，给予Pembrolizumab单药治疗，研究筛选147例患者，PD-L1阳性42例，纳入24例患者，毒性方面：3级以上毒性的发生率为66.7%（16/24），常见的毒性包括关节痛（16.7%）、无力（16.7%）、皮疹（16.7%）、腹泻（12.5%）和疲乏（12.5%），关注的免疫相关的毒性发生率为12.5%（3/24），包括免疫性甲状腺炎，输注部位局部反应，细胞因子释放综合征和结肠炎，与之前Pembrolizumab治疗其他实体瘤的毒性一致；疗效方面：ORR为33.3%，PFS为1.9个月，OS为9.7个月，1年的生存率为37.7%。研究证实Pembrolizumab单药治疗PD-L1阳性经治SCLC具有良好的抗肿瘤活性。Pembro对比拓扑替康治疗复发耐药SCLC的Ⅱ期研究（NCT02963090）将进一步评价Pembro单药在SCLC二线及以上治疗的疗效和安全性。2017年ASCO上报告了pembro维持治疗经4-6周期铂类联合依托泊苷治疗后有效或疾病稳定的ED-SCLC患者的Ⅱ期研究，研究的主要终点为PFS^[12]^。研究纳入45例患者，中位PFS为1.4个月，irPFS为4.7个月。中位OS为9.2个月，疾病控制率为42%；Pembro维持治疗常见的3级以上毒性包括：疲乏、恶心、呼吸困难、转氨酶升高，出现与药物相关严重不良反应为：房室传导阻滞（1例）和糖尿病Ⅰ型（1例），然而与之前CALGB 30504研究中观察组2.1个月和舒尼替尼维持治疗组3.7个月的PFS相比，Pembro维持治疗并没有延缓疾病进展风险，但是Pembro维持治疗的OS与CALGB 30504研究^[13]^中舒尼替尼维持治疗组9.0个月的OS相当，明显优于观察组6.9个月的OS，Pembro维持治疗出现PFS与OS获益不一致的现象，考虑以下因素：其一，免疫靶向药物起效相对缓慢，但作用持久；其二，传统的RECIST评价标准不能准确评价免疫靶向药物的疗效，采用iRECIST进行评价Pembro维持治疗的iPFS为4.7个月，与对OS的影响一致；其三，部分SCLC患者能够从Pembro维持治疗中获益，在该研究中PD-L1表达与疗效的分析发现, 基质中PD-L1表达阳性的患者接受Pembro治疗有获益的趋势，但样本量有限，需要进一步研究。寻找更有效的生物标志物筛选适合进行免疫靶向维持治疗的患者，才能使SCLC真正获益。除了单药外，Pembro联合化疗一线（KEYNOTE-011, REACTION）、二线治疗（PembroPlus, MISP-MK3475）ED-SCLC，Pembro联合放化疗一线治疗SCLC（包括局限期和广泛期）（NCT02402920）以及Pembro联合PI3K抑制剂治疗复发耐药SCLC的研究（NCT02646748）正在进行，将为Pembro在SCLC的治疗中提供更多证据。

### 1.3 PD-L1抑制剂

PD-L1抑制剂Atezolizumab、Durvalumab、Avelumab都有相关研究在SCLC开展。其中最早开始SCLC领域研究的是Atezolizumab。PCD4989g研究^[14]^是一项Atezolizumab治疗包含SCLC在内的多种实体瘤的Ia期研究。起初入组患者需要进行PD-L1筛选，后来研究方案修订，患者入组无需考虑PD-L1状态，研究采用VENTANA PD-L1 (SP142) IHC对肿瘤细胞（tumor cell, TC）和免疫细胞（immune cell, IC）上的PD-L1表达水平进行检测。研究纳入17例SCLC患者，65%的患者既往接受3线以上治疗，Atezoz治疗的相关毒性：11例患者发生治疗相关性不良反应，多数为1级-2级，2例发生3级以上相关性不良反应，1例患者因3级肺炎停药，1例患者发生5级肝功能衰竭。ORR：6%（实体瘤评价标准1.1）；24%（免疫相关反应评价标准），中位PFS：1.5个月（95% CI: 1.2-2.7），中位OS：5.9个月（95% CI: 4.3-20.1）。该研究探索性分析了PD-L1免疫组化（immunohistochemistry, IHC）表达、PD-L1 mRNA表达与PFS、OS的关系，发现PD-L1高表达的患者有更长的irPFS和OS；另外该研究该还探索了效应T细胞（Teff）基因特征与Atezolizumab疗效的关系，发现Teff高表达的患者irPFS和OS更长。初步的结果显Atezo治疗ES-SCLC具有良好的耐受性，单药的疗效令人鼓舞，由于研究纳入的病例较少，与Atezolizumab疗效相关的因素仍需探索。

目前Atezolizumab在SCLC的研究主要集中在一线治疗（NCT02748889, IMpower133, NCT03041311），采用联合治疗的策略。Durvalumab从2016年也开始在SCLC领域开展研究，研究策略是从二线治疗向一线治疗发展，以联合治疗为主，尤其是双免疫联合治疗其他治疗，比如Durvalumab/Tremelimumab联合化疗一线治疗ED-SCLC的Ⅲ期研究（Caspian），Durvalumab/Tremelimumab联合放疗治疗复发SCLC的Ⅱ期研究（NCT02701400），另外Durvalumab联合在DNA修复中发挥重要作用的PARP抑制剂Olaparib治疗实体瘤（包括SCLC）的Ⅰ期/Ⅱ期研究（MEDIOLA）正在如火如荼开展。Avelumab在SCLC领域的研究刚刚开始，仍就是联合策略，期待这些研究结果的公布，为SCLC的治疗提供更多、更有效的治疗选择。

## 2 SCLC免疫靶向治疗的精准探索

与其他肿瘤一样，在精准医学的浪潮下，免疫靶向治疗在SCLC领域同样面临如何实现精准的问题。PD-L1表达是目前研究最广泛的免疫检查点标志物，能否作为标志物筛选适合接受免疫靶向治疗的SCLC也在研究中。KEYNOTE-028研究^[11]^SCLC中PD-L1表达的阳性的患者（≥1%）ORR为33.3%，PCD4989g研究^[11]^发现PD-L1高表达的患者似乎OS更长，而CheckMate 032研究^[5]^并没有发现PD-L1表达与疗效相关。研究者也分析了SCLC中PD-L1的表达情况，Yu等^[15]^对来自波兰格但斯克医科大学的98例局限期SCLC和来自美国社区肿瘤站点的广泛期SCLC标本，采用SP142、Dake28-8两种抗体和检测平台和原位杂记分别检测了PD-L1蛋白表达和mRNA表达。研究通过两种不同的方法和两种不同的抗体检测，发现在两组患者，两种抗体PD-L1≥1%，< 5%分别为11.6%（SP142）、10.4%（Dake28-8）和12.6%（Dake28-8），PD-L1≥5%，< 10%分别为0（SP142）、3.0%（Dake28-8）和0%（Dake28-8），PD-L1≥10%，< 50%分别为2.1（SP142）、3.0%（Dake28-8）和1.1%（Dake28-8），PD-L1≥50%分别为1.1（SP142）、3.0%（Dake28-8）和1.1%（Dake28-8），LD-SCLC组PD-L1 mRNA阳性15.5%，两个独立的SCLC队列中PD-L1的表达情况相似，与非小细胞肺癌（non-small cell lung cancer, NSCLC）相比SCLC中PD-L1的表达较NSCLC低，是否预示SCLC与NSCLC涉及不同的免疫调节机制。而另一项由台湾学者进行的SCLC PD-L1的表达的研究^[16]^，纳入了186例患者，采用Proteintech group公司的PD-L1抗体，PD-L1表达的阳性标准为：≥5%。研究发现肿瘤细胞PD-L1阳性率为78.0%，肿瘤浸润的淋巴细胞PD-L1阳性率为54.3%，认为SCLC高表达PD-L1，PD-L1表达与分期相关，是SCLC的预后因素。这两项研究纳入的人群不同，采用的检测方法不同，研究结果不同，可见SCLC PD-L1检测需要建立统一的标准，也需要探索不同人群是否存在差异。

NSCLC免疫治疗的Checkmate026研究^[17]^证实高突变负荷的患者能够从Nivolumab中获益显著。2017年ASCO一项研究分析了实体瘤的突变负荷^[18]^，研究定义高肿瘤突变负荷（tumor mutation burden, TMB）≥17 mut/Mb，在14种实体瘤中具有高TMB的SCLC（99例）的比例为5%，另一项关于SCLC和大细胞神经内分泌肺癌的TMB和基因改变的研究中发现^[19]^，SCLC TMB的中位数为9 mut/Mb，第90百分位数的TMB为19.6 mut/Mb，但关于SCLC患者TMB与免疫靶向治疗疗效相关性的研究仍缺少相关数据，突变负荷否成为筛选适合的SCLC接受免疫靶向治疗的标志物也是需要探索的问题。另外，最近在对19种恶性肿瘤5, 777个肿瘤标本的全外显子测序发现^[20]^，插入和缺失突变（insertions and deletions mutations, Indel）越多的肿瘤，可能对PD-1抑制剂等免疫治疗更敏感，有效率越高，生存期越长，该研究并未包含SCLC，Indel能否预测SCLC接受免疫靶向治疗的疗效需要进行相关的研究。

最近，Pembrolizumab获批MSI-H（dMMR）全瘤肿，而获批的依据为5项研究的综合，其中包括KEYNOTE-028研究，该研究中有一例存在SCLC MSI-H（dMMR）获得CR，而且缓解持续时间（duration of response, DOR）：8.9 mo+。MSI-H（dMMR）能否成为SCLC免疫治疗的预测标志物，还需要研究SCLC患者存在MSI-H（dMMR）的比例，而且需要更多的研究和数据的支持。SCLC目前正在研究的肿瘤新抗原、免疫组库、肿瘤微环境、TCR等在SCLC免疫治疗中的作用同样需要进一步阐释。

NSCLC的研究中发现存在*P53*基因异常的患者具有更高的肿瘤突变负荷和PD-L1表达^[21]^，从PD-1抑制剂中获益更明显，SCLC普遍存在P53的异常^[22-24]^，然而免疫靶向治疗在SCLC的疗效并未惠及多数患者，仅不超过30%的患者获益，其潜在的机制，影响因素的研究将使SCLC的免疫靶向治疗更精准。

联合治疗是免疫靶向治疗的一种发展趋势，明确与哪些药物联合，怎样联合，不同的联合方案适合哪些特定的SCLC患者才能让SCLC的免疫靶向治疗更精准。研究发现致癌基因*MYC*能够调节肿瘤细胞表面天然免疫调节因子CD47和适应性免疫调节检查点分子PD-L1的表达^[25]^，抑制MYC的活性能够促进CD47和PD-L1的表达，抗肿瘤免疫受到抑制，SCLC大约有6%的患者存在*MYC*基因扩增，这部分SCLC患者采用PD-1/PD-L1抑制剂联合抑制MYC活性的治疗是否更有效？免疫靶向治疗联合靶向DLL3的抗体偶联药物，联合细胞周期检查点激酶WEEK1抑制剂AZD1775的研究，联合PARP抑制剂的研究，将成为针对SCLC发病机制量身定制的免疫治疗。这也提示针对SCLC复发的分子机制探索，将为我们打开精准联合治疗策略的大门。

## 3 免疫检查点之外的免疫治疗在SCLC中的探索

除了目前最火热的针对适应性免疫的免疫检查点抑制治疗，针对天然免疫的治疗也在SCLC进行了探索。CD47是细胞表面分子，是识别自我的一种标志物，通过与表达在巨噬细胞表面的信号调节蛋白α（SIRPα）相互作用，阻止巨噬细胞表达CD47，发挥细胞的吞噬作用^[26, 27]^。在SCLC动物模型阻断CD47促进巨噬细胞吞噬作用，抗CD47抗体治疗SCLC鼠模型能够抑制肿瘤生长，显著延长生存^[28]^。CD47单克隆抗体Hu5F9-G4治疗实体瘤的Ⅰ剂量爬坡研究正在进行（NCT02216409）。

岩藻糖酰单唾液酸神经节苷酯是一种在小细胞肺癌细胞表面特异且大量表达的神经节苷脂^[29]^，能够被NK细胞识别，可以作为治疗的潜在靶点。BMS-986012是单克隆全人源IgG1抗体，可以特异性结合岩藻糖酰单唾液酸神经节苷酯，诱导NK细胞发挥抗肿瘤作用，BMS-986012的Ⅰ期/Ⅱ期研究（CA001-030研究）^[30]^在复发耐药SCLC显示出了良好的安全性并获得了18%的客观缓解，进一步的结果值得期待。

## 4 总结与展望

免疫治疗的进展正在改变肿瘤治疗的临床实践，已经成为黑色素瘤、肾癌、NSCLC、头颈部鳞癌、淋巴瘤、泌尿系肿瘤、默克細胞癌的临床治疗选择。免疫靶向治疗在SCLC中的初步研究结果，让我们对免疫治疗打破SCLC治疗困境的壁垒充满期待。免疫靶向治疗这把利剑在SCLC领域能否披荆斩棘，开辟一条战胜SCLC的希望之路，仍然需要我们对免疫治疗和SCLC分子机制深入的理解。

## References

[1] Vogelstein B, Papadopoulos N, Velculescu VE (2013). Cancer genome landscapes. Science.

[2] Reck M, Bondarenko I, Luft A (2013). Ipilimumab in combination with paclitaxel and carboplatin as first-line therapy in extensive-disease-small-cell lung cancer: results from a randomized, double-blind, multicenter phase 2 trial. Ann Oncol.

[3] Reck M, Luft A, Szczesna A (2016). Phase Ⅲ randomized trial of ipilimumab plus etoposide and platinum versus placebo plus etoposide and platinum in extensive-stage small-cell lung cancer. J Clin Oncol.

[4] Antonia SJ, Lopez-Martin JA, Bendell JC (2016). Checkmate 032: Nivolumab (N) alone or in combination with ipilimumab (Ⅰ) for the treatment of recurrent small cell lung cancer (SCLC). J Clin Oncol.

[5] Antonia SJ, López-Martin JA, Bendell J (2016). Nivolumab alone and nivolumab plus ipilimumab in recurrent small-cell lung cancer (CheckMate 032): a multicentre, open-label, phase 1/2 trial. Lancet Oncol.

[6] O'brien ME, Ciuleanu TE, Tsekov H (2006). Phase Ⅲ trial comparing supportive care alone with supportive care with oral topotecan in patients with relapsed small-cell lung cancer. J Clin Oncol.

[7] von Pawel J, Jotte R, Spigel DR (2014). Randomized phase Ⅲ trial of amrubicin versus topotecan as second-line treatment for patients with small-cell lung cancer. J Clin Oncol.

[8] Hellmann M, Antonia S, Ponce S (2017). MA09. 05 Nivolumab alone or with ipilimumab in recurrent small cell lung cancer (SCLC): 2-year survival and updated analyses from the checkmate 032 trial. J Thorac Oncol.

[9] Kalemkerian GP, Loo BW, Akerley W (2017). NCCN Clinical Practice Guidelines in Oncology. Small Cell Lung Cancer, version 1. 2017.

[10] Hellmann MD, Ott PA, Zugazagoitia J (2016). Nivolumab (nivo) ±ipilimumab (ipi) in advanced small-cell lung cancer (SCLC): First report of a randomized expansion cohort from CheckMate 032. J Clin Oncol.

[11] Ott P, Felip E, Hiret S (2017). OA05.01 Pembrolizumab in patients with extensive-stage small cell lung cancer: updated survival results from KEYNOTE 028. J Thorac Oncol.

[12] Gadgeel SM, Ventimiglia J, Kalemkerian GP (2017). Phase Ⅱ study of maintenance pembrolizumab (pembro) in extensive stage small cell lung cancer (ES-SCLC) patients (pts). J Clin Oncol.

[13] Ready NE, Pang HH, Gu L (2015). Chemotherapy with or without maintenance sunitinib for untreated extensive-stage small-cell lung cancer: a randomized, double-blind, placebo-controlled phase Ⅱ study-CALGB 30504 (Alliance). J Clin Oncol.

[14] Sequist LV, Chiang A, Gilbert J (2016). Clinical activity, safety and predictive biomarkers results from a phase Ia atezolizumab (atezo) trial in extensive-stage small cell lung cancer (ES-SCLC). Ann Oncol.

[15] Yu H, Batenchuk C, Badzio A (2017). PD-L1 expression by two complementary diagnostic assays and mRNA *in situ* hybridization in small cell lung cancer. J Thorac Oncol.

[16] Chang YL, Yang CY, Huang YL (2017). High PD-L1 expression is associated with stage Ⅳ disease and poorer overall survival in 186 cases of small cell lung cancers. Oncotarget.

[17] Carbone DP, Reck M, Paz-Ares L (2017). First-line nivolumab in stage Ⅳ or recurrent non-small-cell lung cancer. N Engl J Med.

[18] Salem ME, Xiu J, Lenz HJ (2017). Characterization of tumor mutation load (TML) in solid tumor. J Clin Oncol.

[19] Chae YK, Tamragouri K, Chung J (2017). Genomic alterations (GA) and tumor mutational burden (TMB) in large cell neuroendocrine carcinoma of lung (L-LCNEC) as compared to small cell lung carcinoma (SCLC) as assessed via comprehensive genomic profiling (CGP). J Clin Oncol.

[20] Turajlic S, Litchfield K, Xu H (2017). Insertion-and-deletion-derived tumour-specific neoantigens and the immunogenic phenotype: a pan-cancer analysis. Lancet Oncol.

[21] Dong ZY, Zhong WZ, Zhang XC (2017). Potential predictive value of TP53 and KRAS mutation status for response to PD-1 blockade immunotherapy in lung adenocarcinoma. Clin Cancer Res.

[22] George J, Lim JS, Jang SJ (2015). Comprehensive genomic profiles of small cell lung cancer. Nature.

[23] Rudin CM, Durinck S, Stawiski EW (2012). Comprehensive genomic analysis identifies SOX2 as a frequently amplified gene in small-cell lung cancer. Nat Genet.

[24] Peifer M, Fernández-Cuesta L, Sos ML (2012). Integrative genome analyses identify key somatic driver mutations of small-cell lung cancer. Nat Genet.

[25] Casey SC, Tong L, Li Y (2016). MYC regulates the antitumor immune response through CD47 and PD-L1. Science.

[26] Weiskopf K, Ring AM, Ho CM (2013). Engineered SIRPα variants as immunotherapeutic adjuvants to anticancer antibodies. Science.

[27] Weiskopf K, Weissman IL (2015). Macrophages are critical effectors of antibody therapies for cancer. MAbs.

[28] Weiskopf K, Jahchan NS, Schnorr PJ (2016). CD47-blocking immunotherapies stimulate macrophage-mediated destruction of small-cell lung cancer. J Clin Invest.

[29] Brezicka FT, Olling S, Nilsson O (1989). Immunohistological detection of fucosyl-GM1 ganglioside in human lung cancer and normal tissues with monoclonal antibodies. Cancer Res.

[30] Chu QC, Markman B, Leighl N (2016). A phase 1/2 trial of a monoclonal antibody targeting fucosyl GM1 in relapsed/refractory small cell lung cancer (SCLC): Safety and preliminary efficacy. Ann Oncol.

